# Enantioselective Synthesis of *cis*-Decalins Using Organocatalysis and Sulfonyl Nazarov Reagents

**DOI:** 10.3390/molecules20046409

**Published:** 2015-04-10

**Authors:** Javier Peña, Gastón Silveira-Dorta, Rosalina F. Moro, Narciso M. Garrido, Isidro S. Marcos, Francisca Sanz, David Díez

**Affiliations:** 1 Departamento de Química Orgánica, Universidad de Salamanca, Plaza de los Caídos 1-5, 37008 Salamanca, Spain; E-Mails: javierpena@usal.es (J.P.); rfm@usal.es (R.F.M.); nmg@usal.es (N.M.G.); ismarcos@usal.es (I.S.M.); 2 Instituto Universitario de Bio-Orgánica “Antonio González” (IUBO-AG), Universidad de La Laguna, Astrofísico Francisco Sánchez 2, 38206 La Laguna, Spain; E-Mail: gsdorta@ull.es; 3 Servicio de Difracción de Rayos X, Plataforma Nucleus, Universidad de Salamanca, Plaza de los Caídos 1-5, 37008 Salamanca, Spain; E-Mail: sdrayosx@usal.es

**Keywords:** organocatalysis, Nazarov reagents, sulfones, decalins

## Abstract

The first organocatalytic synthesis of *cis*-decalins using sulfonyl Nazarov reagents is reported. The Jørgensen’s catalyst directs this highly enantioselective synthesis using different cyclohexenal derivatives.

## 1. Introduction

In the last years there has been a growing interest in organocatalysis [1,2,3,4], a new field which has quickly attracted researchers due to its potential compared to classic catalysis. This methodology has been widely used for the synthesis of natural products [5]. The *cis*-decalin framework is present in the molecular structure of various classes of natural products such as *cis*-clerodanes [6], kalihinenes [7], thelepoganes [8], cadinanes [9], eremophilanes [10], and valeranones [11]. These products have been typically obtained by isolation from natural sources. Many of these *cis*-decalin-based natural products exhibit wide-ranging and interesting biological activities. It is evident that many of these natural products have varying degrees of substitution patterns and four or more contiguous stereogenic centres on the decalin skeleton and, hence, pose a considerable synthetic challenges. The structural complexity of these natural products, together with their interesting biological properties, have led to a significant interest in the development of new and efficient methods for the synthesis of *cis*-decalins in general and the aforementioned natural products in particular [12].

Nazarov reagents have been used for the synthesis of *cis*-decalins based on the so-called Deslongchamps annulation [13], but without control of the absolute stereochemistry [14]. Furthermore there are no examples in literature where sulfonyl Nazarov reagents have been used for the synthesis of *cis*-decalins.

**Scheme 1:**
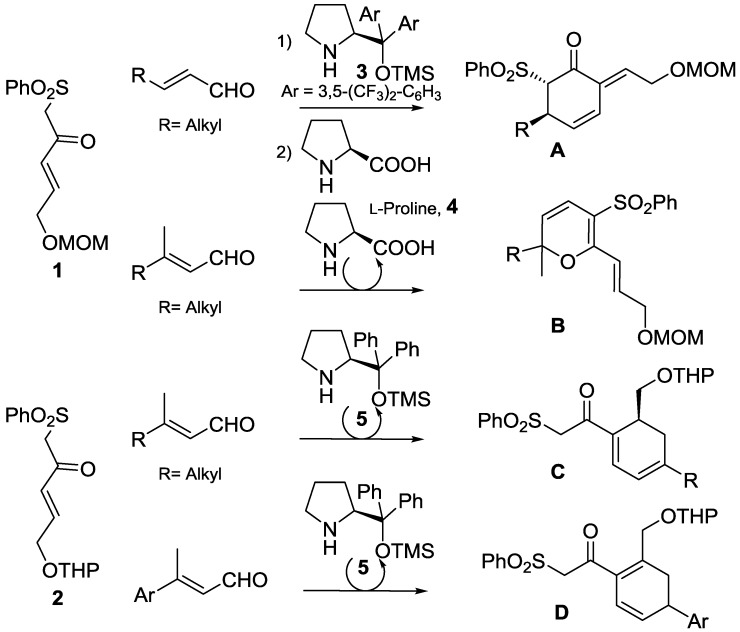
Reactivity of Nazarov reagents **1** and **2** with unsaturated aldehydes.

In the last few years we have studied the reactivity of β-keto-γ,δ-unsaturated sulfones such as **1** and **2** with different organocatalysts and conditions and we have been able to obtain different important scaffolds such as chiral cyclohexenones **A** [15], 2*H*-dihydropyrans **B** [16], and highly functionalised cyclohexa-1,3-dienes **C** and **D** [17] (Scheme 1).

## 2. Results and Discussion

**Scheme 2:**
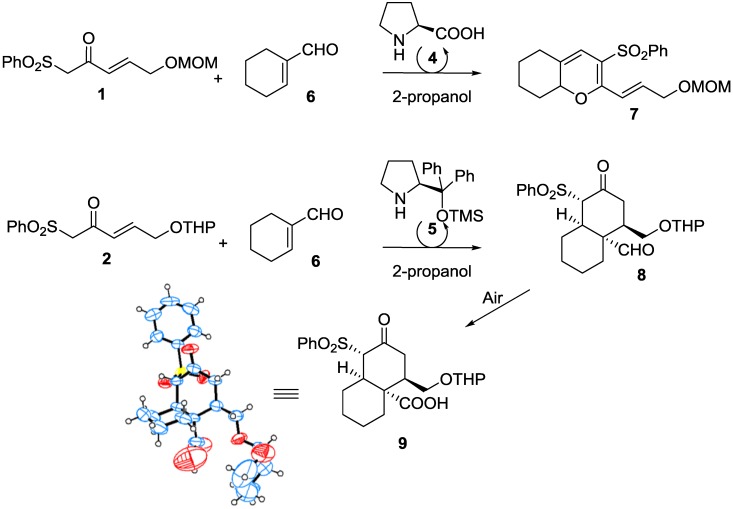
Synthesis of the *cis*-decalin and X-ray crystal structure of compound **9** (displacement ellipsoids are drawn at the 30% probability).

In order to extend these studies towards a diversity oriented synthesis [18], we envisaged the use of our methodology with β-keto-γ,δ-unsaturated sulfones under organocatalytic conditions to synthesize *cis*-decalins using cyclic unsaturated aldehydes as starting materials. Previously we have reacted cyclohexenecarboxaldehyde **6** with **1** in the presence of l-proline to obtain the corresponding 2*H*-dihydropyran **7** [16] (Scheme 2). We then decided to study the reactivity of cyclic unsaturated aldehydes in the presence of the Jørgensen’s catalyst **5**, in order to obtain bicyclic systems. We started evaluating the reactivity of sulfone **2** with enal **6** in 2-propanol, using organocatalyst **5** (Scheme 2). No pyran structure was observed by ^1^H-NMR in this case with **6**, as when proline was used as catalyst. Instead, the ^1^H-NMR spectrum revealed that a new structure that differed from the usual cyclo-hexenones, cyclohexadienes or pyrans had been formed, as now one aldehyde hydrogen and no olefinic hydrogen signals were present in the ^1^H-NMR spectrum. Only one hydrogen signal from the CH_2_ group between sulfonyl and carbonyl groups remained and, as in the case of cyclohexenones, this hydrogen was not coupled with any other, indicating that either there was no hydrogen close to it or that it may be inside a cyclic structure and in a disposition without coupling with proximal hydrogens. We were now also able to see two carbonyl groups in the ^13^C-NMR spectrum, corresponding to a CO and a CHO group. The latter was joined to a tetrasubstituted carbon atom. All these facts made us to think that we had obtained a decalin system such as **8**.

The result was verified by X-ray experiments of the carboxylic acid derivative **9** (Scheme 2) formed by oxidation of aldehyde **8** under normal air atmosphere. This result also corroborated that we had obtained a *cis*-decalin structure [19]. With this result in hand, we examined the effects of different solvents, ratios of starting materials and time on the scope of the reaction (Table 1).

As shown, this reaction does not work in hydrocarbons or ethereal solvents, (entries 1–3). However, in polar protic solvents or even without solvent (entries 4–8), this reaction works affording the corresponding *cis*-decalin **8** in low to moderate yields, being EtOH the solvent which gave the best results. When benzoic acid was used (entry 6), a similar yield was obtained in less time, although products were harder to purify. Moreover, the sulfone/aldehyde ratio was also tested and, as before, the best results to drive the reaction into products are obtained using a 2/1 sulfone/enal ratio. In this study, EtOH proved again to be the best solvent. 

**Table 1:** Solvent and time screening for the reaction of Nazarov reagent **2** with **6**. 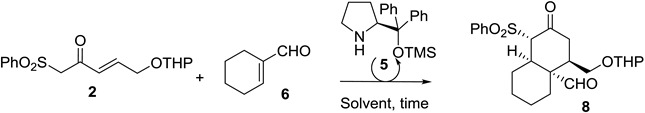

Entry ^[a]^	2/6 ratio.	Solvent	T (h) ^[b]^	Yield [%] ^[c]^	ee
1	2/1	n-Hexane	72	S.M.	--
2	2/1	Et_2_O	72	S.M.	--
3	2/1	THF	72	S.M.	--
4	2/1	MeOH	72	20	ND
5	2/1	EtOH	72	52	96
6 ^[d]^	2/1	EtOH	48	53	85
7	2/1	2-propanol	72	35	ND
8	2/1	H_2_O	48	6	ND
9	2/1	NO SOLVENT	72	18	ND
10	1/1	EtOH	72	30	ND
11	1/2	EtOH	72	42	ND

^[a]^ All the reactions were carried out at rt, in the corresponding solvent at 0.18 M during the specified time using catalyst **5** (20 mol %); ^[b]^ Time in which highest yield was observed with no decomposition (the consumption of starting materials was monitored by TLC); ^[c]^ Isolated yield after chromatography on silica gel; ^[d]^ 20 mol % benzoic acid added. S.M. = Starting material. ND = not determined.

Next we studied the catalyst load for this reaction (Table 2). As shown in Table 2, the reaction does not work without catalyst (entry 1). As the catalyst amount increases, so does the yield (entries 2–5), however, the difference between using 20 or 50 mol % is not enough to justify the increased catalyst load. For this reason, 20 mol % is taken as the optimal catalyst amount to be used for this transformation.

**Table 2:** Catalyst load screening.

Entry ^[a]^	Catalys 5 (%)	Yield [%] ^[b]^	ee
1	0	S.M.	--
2	5	28	ND
3	10	29	ND
4	20	52	96
5	50	60	96

^[a]^ All the reactions were carried out at rt, in EtOH at 0.18 M in 72 h, with a 2/1 ratio of **2**/**6**; ^[b]^ Isolated yield after chromatography on silicagel. S.M. = Starting material.

With the best conditions in hand, we tested different cyclic enals. As shown in Table 3, the reaction also works well with sulfone **1** (entry 3). Moderate results are achieved with other more hindered aldehydes such as (*S*)-(−)-perillaldehyde (**10**, entries 4–9), with the best yield being achieved after adding 20 mol % of benzoic acid. When enantiomeric catalyst *ent*-**5** is used, the reaction works poorly or does not work at all, perhaps because of a mismatched pairing effect between the aldehyde and catalyst substituents (entries 6 and 7). Similar results are obtained with its epoxide **11** (entries 10–12). Reactions with an even more hindered aldehyde such as (1*R*)-myrtenal (**12**) or with smaller pentacyclic enal **13** (entries 15–19) did not work.

**Table 3:** Reaction of Nazarov reagents **1** and **2** with cyclic enals ^[a]^. 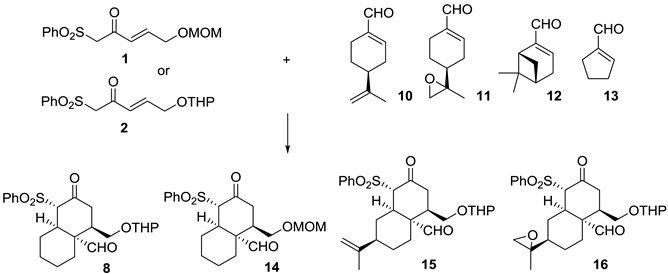

Entry ^[a]^	Cyclic Enal.	Product	T (h) ^[b]^	Yield [%] ^[c]^	ee ^[d]^
1	**6**	**8**	72	51	96
2 ^[e]^	**6**	***ent*-8**	72	51	-96
3 ^[f,g]^	**6**	**14**	48	50	96
4	**10**	**15**	48	63	90
5	**10**	**15**	96	15	N.D.
6 ^[g]^	**10**	**15**	48	85	90
7 ^[e]^	**10**	***ent*-15**	48	4	N.D.
8 ^[e]^	**10**	--	72	--	N.D.
9 ^[h]^	**10**	***ent*-15**	96	4	N.D.
10	**11**	**16**	48	22	N.D.
11 ^[i]^	**11**	**16**	48	39	-- ^[j]^
12 ^[g,i]^	**11**	**16**	48	30	N.D.
13	**12**	S.M.	96	--	--
14 ^[e]^	**12**	S.M.	120	--	--
15	**13**	S.M. ^[k]^	48	--	--
16	**13**	S.M. ^[k]^	72	--	--
17	**13**	S.M. ^[k]^	96	--	--
18	**13**	S.M. ^[k]^	120	--	--
19 ^[g]^	**13**	S.M. ^[k]^	120	--	--

^[a]^ All the reactions were carried out at rt, in EtOH at 0.18 M during the specified time, with a 2/1 ratio of **2**/**cyclic enal** and catalyst **5** (20 mol %); ^[b]^ Time in which highest yield was observed with no decomposition (the consumption of starting materials was monitored by TLC); ^[c]^ Isolated yield after chromatography on silica gel; ^[d]^ ee determined by HPLC analysis, carried out on a CHIRALPAK IC column; ^[e]^ ***ent*-5** (20 mol %); ^[f]^ Sulfone **1** (1 equiv.) used; ^[g]^ 20 mol % benzoic acid added; ^[h]^ ***ent*-5** (50 mol %); ^[i]^ **5** (50 mol %); ^[j]^ Complex HPLC results were obtained and we are currently working on these results; ^[k]^ Only starting sulfone **2** was recovered; S.M. = Starting material. N.D. = Not determined.

**Scheme 3:**
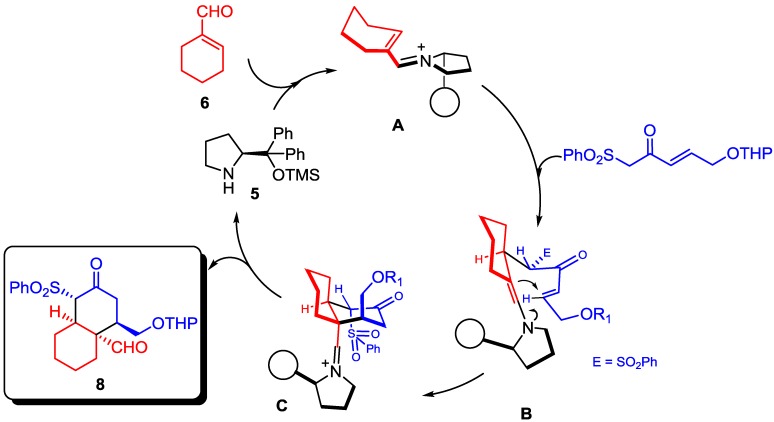
Proposed mechanism for the synthesis of cis-decalins.

The Diels-Alder mechanism proposed by Deslogchamps [14] does not explain the stereochemical outcome of our procedure since, according to this pathway, neither the *endo*- nor the *exo*-approach produce the same stereochemistry. These results can be explained by a Diels-Alder mechanism only if the configuration of any of the double bonds in the diene is *cis*, what seems to be quite unlikely. Hence, we propose the double-Michael mechanism depicted in Scheme 3, as proposed by Deslongchamps too [20]. First, dienamine **A** is formed between the catalyst and the α,β-unsaturated aldehyde, then the Nazarov reagent acts as nucleophile forming **B**. This enamine reacts with the α,β-unsaturated ketone affording **C** which after elimination of catalyst yields bicycle **8** with the stereochemistry observed by X-ray experiments.

## 3. Experimental Section

### 3.1. General

^1^H-NMR and ^13^C-NMR spectra were recorded in CDCl_3_ at 200 and 400 MHz (^1^H) or 50 and 100 MHz (^13^C) on Varian 200 VX (Salamanca, Spain) and Bruker DRX 400 instruments (Salamanca, Spain), respectively. Multiplicities were determined by DEPT experiments. IR spectra were registered using a BOMEM (Salamanca, Spain) 100 FTIR spectrophotometer. Optical rotations were determined using a Perkin-Elmer 241 polarimeter in a 1 dm cell and are given in units of 10^−1^ deg cm^2^ g^−1^. Concentrations are quoted in g per 100 mL. The electron impact (EI) mass spectra were run on a VG-TS 250 spectrometer (Salamanca, Spain) using a 70 eV ionizing voltage. HRMS were recorded using a VG Platform (Fisons, Salamanca, Spain) spectrometer using Chemical Ionization (ammonia as gas) or Fast Atom Bombardment (FAB) techniques. Thin layer chromatography (tlc) was performed on aluminum sheets coated with 60 F254 silica. Sheets were visualized using iodine, UV light or 1% aqueous KMnO_4_ solution. Column chromatography (CC) was performed with Merck silica gel 60 (70–230 mesh). Solvents and reagents were generally distilled prior to use. Dichloromethane (DCM) was distilled from KOH.

### 3.2. General Procedure for the Synthesis of Chiral cis-Decalins **8**, **14**‒**16**

β-Ketosulfone **1** or **2** (0.15 mmol) and the corresponding aldehyde **6** (0.07 mmol) were dissolved in EtOH (1 mL). Next, catalyst **5** (20 mol %) was added and the mixture was left to stir at room temperature for 48–72 h. After concentrating under vacuum, the residues were purified by flash chromatography on silica gel using different mixtures of *n*-hexane/EtOAc. Note: for the synthesis of enantiomeric derivatives catalyst *ent***-5** was used instead.

#### 3.2.1. (1*S*,4*R*,4a*S*,8a*R*)-2-Oxo-1-(phenylsulfonyl)-4-(((tetrahydro-2*H*-pyran-2-yl)oxy)methyl)-deca-hydronaphthalene-4a-carbaldehyde (**8**)

Note: the presence of THP protecting group makes many of NMR signals, both ^1^H and ^13^C, to appear twice. Hence, for compounds with THP groups only the NMR shift values for one isomer are given here. Colorless oil (15.3 mg, 51%): ν*_max_* (liquid film) 2939, 2868, 1716, 1448, 1321, 1309, 1149; δ_H_ (200 MHz; CDCl_3_) 9.63 (1H, s, CHO), 7.86 (2H, d, *J* = 7.2 Hz, ArH*_ortho_*), 7.70 (1H, d, *J* = 6.9 Hz, ArH*_para_*), 7.66–7.51 (2H, m, *J* = 7.2 Hz, ArH*_meta_*), 4.51 (1H, d, *J* = 12.4 Hz, H24), 3.82–3.64 (2H, m, H1 and H26_A_), 3.62–3.36 (3H, m, H8a and H1'), 3.26–3.10 (1H, m, H26_B_), 3.02–2.70 (2H, m, H3), 2.23–1.92 (2H, m, H4 and H5_A_), 1.66–1.48 (13H, m, H27, H28, H29, H5_B_, H6, H7 and H8); δ_C_ (50 MHz; CDCl_3_) 203.8 (CH, CHO), 202.2 (C, C=O), 137.3 (C, ArC*_ipso_*), 134.7 (CH, ArC*_para_*), 129.5 (2CH, ArC*_meta_*), 129.2 (2CH, ArC*_ortho_*), 98.9 (CH, C24), 75.4 (CH, C1), 66.6 (CH_2_, C1'), 62.3 (CH_2_, C26), 50.0 (C, C4a), 39.8 (CH, C4), 37.8 (CH_2_, C3), 35.6 (CH, C8a), 30.4 (CH_2_, C29), 30.2 (CH_2_, C5), 25.5 (CH_2_, C27), 22.2 (CH_2_, C8), 21.4 (CH_2_, C7), 19.7 (CH_2_, C6), 19.1 (CH_2_, 28); EIHRMS: Calcd. for C_23_H_30_O_6_S (M+Na): 457.1661; found 457.1655 (M+Na); er: determined by HPLC: CHIRALPAK IC column; *n*-hexane/2-propanol [60/40 (v/v)]; flow rate: 1.0 mL/min; λ = 210 nm t_R_ (minor) = 19.1, 20.8 min; t_R_ (major) = 26.5, 37.5 min; ${\left[\alpha \right]}_{D}^{25}$ = −12.6 (c = 2. 65, CHCl_3_).

#### 3.2.2. (1*R*,4*S*,4a*R*,8a*S*)-2-Oxo-1-(phenylsulfonyl)-4-(((tetrahydro-2*H*-pyran-2-yl)oxy)methyl)deca-hydronaphthalene-4a-carbaldehyde (*ent*-**8**)

Colorless oil (15.3 mg, 51%): ${\left[\alpha \right]}_{D}^{25}$ = +12.9 (c = 1.54, CHCl_3_).

#### 3.2.3. (1*S*,4*R*,4a*S*,8a*R*)-4-((Methoxymethoxy)methyl)-2-oxo-1-(phenylsulfonyl)octahydronaphthalene-4a(2*H*)-carbaldehyde (**14**)

Colorless oil (151 mg, 60%): ν*_max_* (liquid film) 2937, 2868, 1718, 1448, 1321, 1307, 1149; δ_H_ (200 MHz; CDCl_3_) 9.61 (1H, s, CHO), 7.96–7.78 (2H, m, ArH*_ortho_*), 7.67–7.53 (3H, m, ArH*_meta_* and ArH*_para_*), 4.51 (2H, s, O-CH_2_-O), 3.71 (1H, d, *J* = 4.7 Hz, H1), 3.52–3.35 (3H, m, H8a and H1'), 3.32 (3H, s, CH_3_-O), 3.05–2.76 (2H, m, H3), 2.24–1.97 (2H, m, H4 and H5_A_), 1.81–1.33 (7H, m, H5_B_, H6, H7 and H8); δ_C_ (50 MHz; CDCl_3_) 203.7 (CH, CHO), 202.1 (C, C=O), 137.2 (C, ArC*_ipso_*), 134.8 (CH, ArC*_para_*), 129.6 (2CH, ArC*_meta_*), 129.2 (2CH, ArC*_ortho_*), 96.6 (CH_2_, O-CH_2_-O), 75.3 (CH, C1), 66.9 (CH_2_, C1'), 55.9 (CH_3_, CH_3_-O), 50.1 (C, C4a), 39.4 (CH, C4), 37.9 (CH_2_, C3), 35.7 (CH, C8a), 30.2 (CH_2_, C5), 22.2 (CH_2_, C8), 21.3 (CH_2_, C7), 19.8 (CH_2_, C6); EIHRMS: Calcd. for C_20_H_26_O_6_S (M+Na): 417.1348; found: 417.1242 (M+Na); ee: determined by HPLC: CHIRALPAK IC column; *n*-hexane/2-propanol [60/40 (v/v)]; flow rate: 1.0 mL/min; λ = 210 nm t_R_ (minor) = 16.8 min; t_R_ (major) = 39.5 min; ${\left[\alpha \right]}_{D}^{25}$ = −8.66 (c = 0.75, CHCl_3_).

#### 3.2.4. (1*S*,4*R*,4a*S*,7*R*,8a*R*)-2-Oxo-1-(phenylsulfonyl)-7-(prop-1-en-2-yl)-4-(((tetrahydro-2*H*-pyran-2-yl)oxy)methyl)decahydronaphthalene-4a-carbaldehyde (**15**)

Colorless oil (15.3 mg, 51%): ν*_max_* (liquid film) 2939, 2870, 1714, 1448, 1319, 1309, 1149; δ_H_ (200 MHz; CDCl_3_) 9.63 (1H, s, CHO), 7.96–7.79 (2H, m, ArH*_ortho_*), 7.78–7.49 (3H, m, ArH*_meta_* and ArH*_para_*), 4.64 (2H, d, *J* = 15.5 Hz, H2''), 4.51 (1H, d, *J* = 12.2 Hz, H24), 3.86–3.64 (2H, m, H1 and H26_A_), 3.64–3.44 (3H, m, H8a and H1'), 3.27–3.11 (1H, m, H26_B_), 3.11–2.65 (2H, m, H3), 2.29–1.99 (2H, m, H4 and H5_A_), 1.66–1.48 (15H, m, H27, H28, H29, H5_B_, H6, H7, H8 and CH_3_-C1''); δ_C_ (50 MHz; CDCl_3_) 203.5 (CH, CHO), 202.1 (C, C=O), 148.2 (C, C1''), 137.2 (C, ArC*_ipso_*), 134.8 (CH, ArC*_para_*), 129.6 (2CH, ArC*_meta_*), 129.2 (2CH, ArC*_ortho_*), 109.9 (CH_2_, C2''), 98.9 (CH, C24), 75.9 (CH, C1), 66.7 (CH_2_, C1'), 62.1 (CH_2_, C26), 49.7 (C, C4a), 39.5 (CH, C4), 37.7 (CH_2_, C3), 36.1 (CH, C7), 35.3 (CH, C8a), 30.4 (CH_2_, C29), 30.2 (CH_2_,C5), 25.5 (CH_2_, C27), 22.4 (CH_2_, C8), 22.3 (CH_2_, C6), 20.9 (CH_3_, CH3-C1''), 22.2 (CH_2_, C6), 19.1 (CH_2_, 28); EIHRMS: Calcd. for C_26_H_34_O_6_S (M+Na): 497.1974; found 497.1968 (M+Na); ee: determined by HPLC: CHIRALPAK IC column; *n*-hexane/2-propanol [60/40 (v/v)]; flow rate: 1.0 mL/min; λ = 210 nm t_R_ (major) = 19.7, 25.5 min; t_R_ (minor) = 46.6, 53.2, 61.4 min; ${\left[\alpha \right]}_{D}^{25}$ = +17.5 (c = 3.4, CHCl_3_).

#### 3.2.5. (1*S*,4*R*,4a*S*,7*R*,8a*R*)-7-(2-Methyloxiran-2-yl)-2-oxo-1-(phenylsulfonyl)-4-(((tetrahydro-2*H*-pyran-2-yl)oxy)methyl)decahydronaphthalene-4a-carbaldehyde (**16**)

Colorless oil (13.4 mg, 39%): ν*_max_* (liquid film) 3478, 2941, 2872, 1720, 1660, 1447, 1321, 1309, 1150; δ_H_ (200 MHz; CDCl_3_) 9.62 (1H, s, CHO), 8.02–7.79 (2H, m, ArH*_ortho_*), 7.79–7.52 (3H, m, ArH*_meta_* and ArH*_para_*), 4.51 (1H, d, *J* = 14.6 Hz, H24), 3.81–3.41 (5H, m, H1, H26_A_, H1' and H8a), 3.25–3.09 (1H, m, H26_B_), 2.95–2.71 (2H, m, H3), 2.50 (2H, s, H2''), 2.27–2.03 (2H, m, H4 and H5_A_), 1.81–1.40 (m, 12H), 1.14 (3H, s, CH_3_-C1''). EIHRMS: Calcd. for C_26_H_34_O_7_S (M+Na): 513.1923; found 513.1917 (M+Na). ee: determined by HPLC: CHIRALPAK IC column; *n*-hexane/2-propanol [80/20 (v/v)]; flow rate: 1.0 mL/min; λ = 290 nm t_R_ = 26.9, 29.0, 33.3, 35.8, 45.9, 49.7 min; ${\left[\alpha \right]}_{D}^{25}$ = −0.3 (c = 2.3, CHCl_3_).

#### 3.2.6. X-ray Crystal Data for Compounds **9**

A suitable single crystal of **9** compound was mounted on glass fibre for data collection on a Bruker Kappa APEX II CCD diffractometer. Data were collected at 298(2) K using Cu K_α_ radiation (λ = 1.54178 Å) and ω scan technique, and were corrected for Lorentz and polarization effects. Structure solution, refinement and data output were carried out with the SHELXTLTM (19) program package. The structure was solved by direct methods combined with difference Fourier synthesis and refined by full-matrix least-squares procedures, with anisotropic thermal parameters in the last cycles of refinement for all non-hydrogen atoms. H(10) and H(16) atoms of SP^3^ hybridized carbons were located directly in a difference Fourier map and freely refined. The rest of the hydrogen atoms were positioned geometrically. Crystal data for **9**: C_23_H_30_O_7_S, M = 450.53, monoclinic, space group C2 (nº 5), a = 21.294(3) Å, b = 6.6141(10) Å, c = 16.324(2) Å, α = γ = 90°, β = 105.491(10)°, V = 2215.5(5) Å^3^, Z = 4, D_c_ = 1.351 Mg/m^3^, m = (Cu-K_α_) = 1.658 mm^−1^, F(000) = 960. 4599 reflections were collected at 2.81 ≤ 2θ ≤ 66.38 and merged to give 2754 unique reflections (R_int_ = 0.0520), of which 2144 with I > 2 σ(I) were considered to be observed. Final values are R = 0.1035, *w*R = 0.3323, GOF = 1.311, max/min residual electron density 0.474 and −0.429 e·Å^−3^. Crystallographic data (excluding structure factors) for the structure reported in this paper have been deposited at the Cambridge Crystallographic Data Centre as supplementary material nº. CCDC 1050978.

## 4. Conclusions

We have disclosed for the first time how *cis*-decalins can be prepared from a sulfonyl Nazarov reagent by a mechanism other than a Diels-Alder reaction under organocatalytic and environmentally safe conditions. This procedure affords polysubstituted *cis*-decalins in moderate to good yields and good to excellent enantioselectivities, opening a new way for the synthesis of many natural occurring products with important biological activities.
